# Antitumor effects of β-elemene via targeting the phosphorylation of insulin receptor

**DOI:** 10.1530/ERC-18-0370

**Published:** 2018-11-12

**Authors:** Dawei Wu, Dongwei Lv, Ting Zhang, Lianying Guo, Fangli Ma, Caihua Zhang, Guofeng Lv, Lin Huang

**Affiliations:** 1 Department of Pathophysiology, College of Basic Medical Sciences, Dalian Medical University, Dalian, Liaoning, China; 2 Department of Sports Medicine, College of Basic Medical Sciences, Dalian Medical University, Dalian, Liaoning, China

**Keywords:** β-elemene, antitumor therapy, insulin receptor signaling, insulin-like growth factor 1 receptor blockade

## Abstract

Ewing sarcoma family tumors (ESFTs) are a group of aggressive and highly metastatic tumors lacking efficient therapies. Insulin-like growth factor 1 receptor (IGF1R) blockade is one of the most efficient targeting therapy for ESFTs. However, the appliance is obstructed by drug resistance and disease recurrence due to the activation of insulin receptor (IR) signaling induced by IGF1R blockade. Herein β-elemene, a compound derived from natural plants, exhibited a remarkable proliferation repression on ESFT cells, which was weakened by a caspase inhibitor Z-VAD. β-elemene in combination with IGF1R inhibitors enhanced markedly the repression on cellular proliferation and mTOR activation by IGF1R inhibitors and suppressed the PI3K phosphorylation induced by IGF1R inhibitors. To investigate the mechanisms, we focused on the effects of β-elemene on IR signaling pathway. β-elemene significantly suppressed the insulin-driven cell growth and the activation of mTOR and PI3K in tumor cells, while the toxicity to normal hepatocytes was much lower. Further, the phosphorylation of IR was found to be suppressed notably by β-elemene specifically in tumor cells other than normal hepatocytes. In addition, β-elemene inhibited the growth of ESFT xenografts *in vivo*, and the phosphorylation of IR and S6 ribosomal protein was significantly repressed in the β-elemene-treated xenografts. These data suggest that β-elemene targets IR phosphorylation to inhibit the proliferation of tumor cells specifically and enhance the effects of IGF1R inhibitors. Thus, this study provides evidence for novel approaches by β-elemene alone or in combination with IGF1R blockades in ESFTs and IR signaling hyperactivated tumors.

## Introduction

Ewing sarcoma family tumors (ESFTs) are a group of aggressive and highly metastatic tumors predominantly afflicting children and young adults. With conventional treatments including chemotherapy, radiotherapy and a multimodal therapy of surgical resection associated with local radiotherapy and chemotherapy, the long-term survival rate for patients with localized tumors is only 70% and that for individuals with metastases is less than 20%. Thus, novel treatments are needed urgently.

The reciprocal chromosomal translocation t (11;22) (q24;q12) is found in 85% of ESFTs, which leads to the fusion between the 5′ segment of the Ewing sarcoma breakpoint region 1 gene (*EWSR1*) and the 3′ portion of Friend leukemia virus integration site 1 gene (*FLI1*). The resulting EWS-FLI1 fusion protein induces insulin-like growth factor 1 (IGF1) expression and suppresses the expression of insulin-like growth factor-binding protein 3 (IGFBP3) (Prieur *et al*. 2004, France *et al*. 2011), therefore enhancing the IGF1 signaling in ESFTs. Hyperactivated IGF1 signaling contributes to the ESFT tumorigenesis. High levels of circulating IGF1 are also associated with the development of several other tumors, including breast cancers, prostate cancers and sarcomas (Renehan *et al*. 2004). Downregulation of IGF1 receptor (IGF1R) expression or blocking IGF1R signaling leads to tumor growth suppression (Sachdev & Yee 2007, Ryan & Goss 2008) and increases the susceptibility of tumor cells to chemotherapies (Tao *et al*. 2007, Chitnis *et al*. 2008, Ryan & Goss 2008). IGF1R targeting therapies exhibit remarkable activity toward ESFTs in clinical studies (Kurzrock *et al*. 2010, Olmos *et al*. 2010). However, the use of IGF1R blockade in patients is associated with hyperinsulinemia which stimulates the proliferation of epithelial cells (Gualberto & Pollak 2009, Pollak 2012). The holo-insulin receptor (IR) formation and the signaling of IR pathway is activated by IGF1R depletion (Zhang *et al*. 2007), and overexpression of IR and IGF1 may result in resistance to IGF1R-targeting therapies (Zhang *et al*. 2007, Hendrickson & Haluska 2009). IR exists in two splice variant isoforms, IR-A and IR-B, which could be activated by insulin (Sciacca *et al*. 2003). In addition, IGF2 can activate IR-A other than IR-B (Sciacca *et al*. 2002). Meanwhile, lots of ESFT patients develop resistance to IGF1R targeting and disease recurrence within several months (Toretsky & Gorlick 2010), and a major mechanism may involve increased IR-A expression, enhanced IR homodimer formation and an activation of IR signaling pathways (Garofalo *et al*. 2011, Lamhamedi-Cherradi *et al*. 2016). Notably, the IR-knockout tumors exhibited more sensitivity to anti-IGF1R therapy (Ulanet *et al*. 2010). Therefore, inhibition of both IR and IGF1R signaling may enhance therapeutic efficacy against IGF1-driven cancers (Sachdev & Yee 2007). However, the IR blockade was considered to be too dangerous due to the significant impairment on glucose metabolism.

β-elemene (1-methyl-1-vinyl-2, 4-diisopropenyl-cyclohexane), a compound derived from natural plants, including lemon grass and *Curcuma wenyujin* plant, has been clinically used to treat several kinds of tumors. β-elemene inhibits proliferation, induces apoptosis, reverses the drug resistance and enhances chemotherapeutic sensitivity of cancer cells (Li *et al*. 2013,^ 2016^, Wu *et al*. 2017). However, the molecular mechanisms in detail remain largely unknown. In addition, the effects of β-elemene on ESFTs are to be determined. In this study, we find that β-elemene represses the proliferation of ESFT cells, enhances the anti-growth effects of IGF1R inhibitors on ESFT cells and decreases the phosphorylation of IR in tumor cells other than normal hepatocytes. Thus, this study provides the evidence for β-elemene alone or in combination with IGF1R blockades as novel treatments for ESFTs and IR signaling hyperactivated tumors.

## Materials and methods

### Cell lines and cell culture

The ESFT cell line A673, the heptacellular carcinoma cell line HepG2, the normal liver cell line THLE2 and the melanoma cell line A2058 were purchased from ATCC, and MHH-ES-1 was purchased from Deutsche Sammlung von Mikroorganismen und Zellkulturen (DSMZ, Germany). These cells were tested by the cell banks for eight STR loci and the *amelogenin* gene. A673, MHH-ES-1 and A2058 were grown in DMEM supplemented with 10% fetal bovine serum (FBS). HepG2 was grown in MEM supplemented with 10% FBS. THLE2 was grown in Bronchial Epithelial Cell Growth Medium (BEGM) supplemented with 5 ng/mL EGF, 70 ng/mL phosphoethanolamine and 10% FBS. The flasks used for THLE2 were precoated with a mixture of 0.01 mg/mL fibronectin, 0.03 mg/mL bovine collagen type I and 0.01 mg/mL bovine serum albumin dissolved in BEBM medium. All the cell lines were incubated at 37°C with 5% CO_2_. Cell lines purchased were passaged less than 30 passages after resuscitation.

### Chemicals

β-elemene (95%) was obtained from Yuanda Pharmaceuticals (Dalian, China). Insulin was from Prospec (Israel). Picropodophyllin (PPP) was purchased from Sellek. NVP-AEW541 and Z-VAD were purchased from MedChem Express (Monmouth Junction, NJ, USA).

### Plasmid

The human WT *INSR* (HIR WT) expression plasmid which includes the full sequence of *INSR* gene and encodes IR-B was a gift from Dr Frederick Stanley (Addgene plasmid # 24049) (Jacob *et al*. 2002).

### Cell viability assay

Cells were seeded at 5 × 10^3^ cells /well into 96-well plates in triplicate. After 24 h, β-elemene was added at the concentration indicated. Forty eight hours later, cell proliferation was determined using Cell Counting Kit-8 (mixture of WST-8 and 1-Methoxy PMS) (Dojindo, Kumamoto, Japan) according to the manufacturer’s instructions. Absorbance was measured at 450 nm with the reference at 630 nm. Cell viability was calculated using the formula:


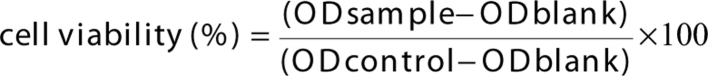


### Colony formation assay

Cells were seeded at 1.2 × 10^3^/well into 6-well plates and were exposed to various concentrations of β-elemene. After 24 h, cells were washed with PBS and supplemented fresh medium. Two weeks later, the colonies were stained with 0.1% crystal violet. The colonies with diameter >2 mm were counted.

### Apoptosis assay

Apoptosis was measured by fluorescence-activated cell sorter using the Annexin V- FITC Apoptosis Detection Kit (Thermo Fisher). In brief, cells plated in six-well plates were treated with β-elemene. After treatment of 24 h, cells were collected and washed once with cold PBS, and subsequently stained simultaneously with FITC-labeled annexin V and PI. Stained cells were analyzed using Accuri C6 (BD).

### Antibodies and Western blot analysis

Antibodies were purchased for the detection of β-actin (AC-15; Sigma); PRAS40 (Invitrogen); cleaved PARP, p-Akt (S473), p-PDK1, p-PRAS40 (T246), p-PI3K (p85), p-S6 ribosomal protein, p-mTOR (S2448), S6 ribosomal protein, p-IGF1R (Y1135/1136)/p-IR (Y1150/1151), S6, Akt, PI3K (p85), mTOR, IGF1R and IR (Cell Signaling); p-IR (Y1361, Abcam); p-ERK1/2 and ERK1/2 (Abbkine). Western blot analysis was performed as described previously (Huang *et al*. 2014), and the signals were detected using an ECL Plus Detection System (Thermo Fisher). Images were acquired using an Image Analyzer ChemiDoc XRS+ (Bio-Rad) and quantified with Image J software.

### *In vivo* tumorigenicity assay

All animal maintenance and procedures were carried out in strict accordance with the recommendations established by the Animal Care and Ethics Committee of Dalian Medical University. The protocol was approved by the Animal Care and Ethics Committee of Dalian Medical University. In animal study, all efforts were made to minimize suffering of mice.

All animals were maintained and animal experiments were conducted in the specific-pathogen-free Laboratory Animal Center of Dalian Medical University. A673 cells (5 × 10^6^) were injected subcutaneously into the two posterior flanks of male BALB/c nude mice (Dalian Medical University). Tumors were allowed to grow for 1 week when the initial measurement was made with calipers. The mice without tumor formation or with a tumor volume 50% bigger than the average were removed from the experiments. The mice were randomly divided into control, low (50 mg/kg) and high doses (100 mg/kg) group, and β-elemene was injected to peritumoral region once per day for up to 17 days (*n* = 12/group). Tumors were measured with a caliper every 2 days, and the tumor volume was calculated using the formula *V* = 1/2 (width^2^ × length). Body weights were also recorded. All mice were killed on day 18, and the tumors were dissected, weighed and measured.

### Immunohistochemistry

Formalin-fixed, paraffin-embedded xenograft tumor sections were deparaffinized, dehydrated and treated with 0.3% hydrogen peroxide. Slides were incubated with anti-p-IR (Abcam) or anti-p-S6 ribosomal protein (Cell Signaling) antibodies overnight at 4°C followed by incubation with biotinylated secondary antibodies (Vector Laboratories, Burlingame, CA, USA) for 1 h at room temperature. Signals were detected using a diaminobenzidine substrate kit (Vector Laboratories). Slides were counterstained with hematoxylin.

The degree of staining was interpreted semiquantitatively by assessing the intensity and extent of staining for each slide. The percent area of positively staining analyzed with ImageJ software was multiplied by their degree of staining (none (0), weakly (1), moderate (2), strong (3)). A staining score (H-score) was then calculated (out of a maximum of 300) (Bollag *et al*. 2010).

### Statistical analyses

All experiments were repeated thrice. The data are represented as the mean ± standard deviation (s.d.). Differences between groups were assessed by one-way ANOVA or Student’s *t*-test. *P* < 0.05 was considered statistically significant. SPSS 17.0 software was used for all statistical analyses.

## Results

### β-elemene represses the proliferation of ESFT cells

**Figure 1:**
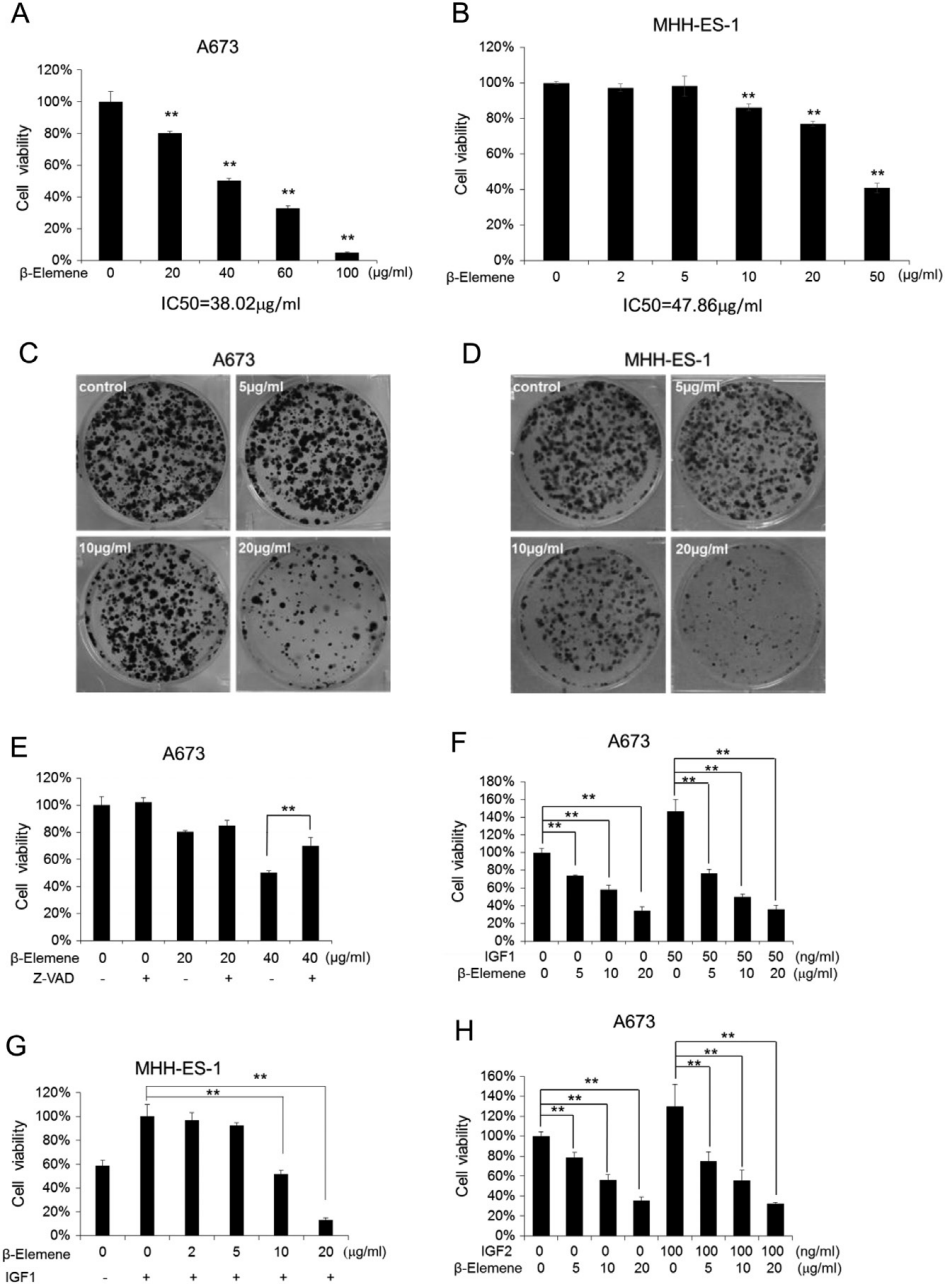
Effects of β-elemene on cellular proliferation. (A and B) Cell viability assays for A673 (A), MHH-ES-1 (B) cells. (C and D) Colony formation assays for A673 (C) and MHH-ES-1 (D) cells. (E) Cell viability assays for A673 cells with increasing concentrations of β-elemene and Z-VAD treatment. Z-VAD, 50 µM. (F, G and H) Cell viability assays for A673 (F and H) and MHH-ES-1 (G) cells in response to IGF1 (F and G) or IGF2 (H) stimulation. The cells were treated with or without (−) IGF1 (50 ng/mL) or IGF2 (100 ng/mL) and increasing concentrations of β-elemene after 24-h starvation. All the experiments were conducted in triplicate, and three independent experiments were performed. ** *P* < 0.01 indicates the significant difference from the untreated cells (A and B), or the particular treated cells as labeled (E, F, G and H). Bars, s.d.

To investigate the effects of β-elemene on the proliferation of ESFT cells, we treated A673 cells with increasing concentrations of β-elemene and evaluated the cellular proliferation with a cell viability assay. The cells showed a significant decline in viability compared with the control in a β-elemene-dose-dependent manner (*P* < 0.01), IC50 is 38.02 µg/mL (Fig. 1A). MHH-ES-1 cells, another ESFT cell line, exhibited a similar reduction as A673 cells in cell viability under β-elemene treatment compared with control (*P* < 0.01), IC50 is 47.86 µg/mL (Fig. 1B). Consistent with the cellular proliferation inhibition, β-elemene also significantly repressed the colony formation in both A673 and MHH-ES-1 cells in a dose-dependent manner (Fig. 1C and D).

To clarify whether the cell growth repression by β-elemene is due to inducing apoptosis, we selected a caspase inhibitor Z-VAD, which inhibits cell apoptosis to treat A673 cells together with β-elemene. The results showed that Z-VAD treatment restored the decreased cell viability by β-elemene (40 µg/mL) from 50 to 70% (*P* < 0.01), which suggests that β-elemene treatment may induce the apoptosis of ESFT cells (Fig. 1E).

IGF1 plays an important role in the proliferation of ESFT cells, and IGF1 was found to be able to increase the cell growth to 1.5- to 1.7-folds compared with control. To test the anti-proliferative activity of β-elemene in the ESFT cells that were stimulated with exogenous IGF1, both A673 and MHH-ES-1 cells were treated with IGF1 and increasing concentrations of β-elemene. β-elemene (5–10 µg/mL) downregulated the exogenous IGF1-driven cell growth to the level of that without IGF1 treatment (Fig. 1F and G). The data showed that β-elemene remarkably inhibited the IGF1-driven ESFT cell growth dose dependently (*P* < 0.01). IGF2 production was previously reported to be increased in IGF1 blockade-resistant cell lines (Garofalo *et al*. 2011). Significant repressive effects on IGF2-driven cell growth as same as IGF1-driven cell growth by β-elemene treatment was confirmed (Fig. 1H) (*P* < 0.01). The data suggest that either IGF1- or IGF2-driven ESFT cell growth is sensitive to β-elemene treatment.

### β-elemene enhances the toxicity of IGF1R inhibitors on ESFT cells

**Figure 2:**
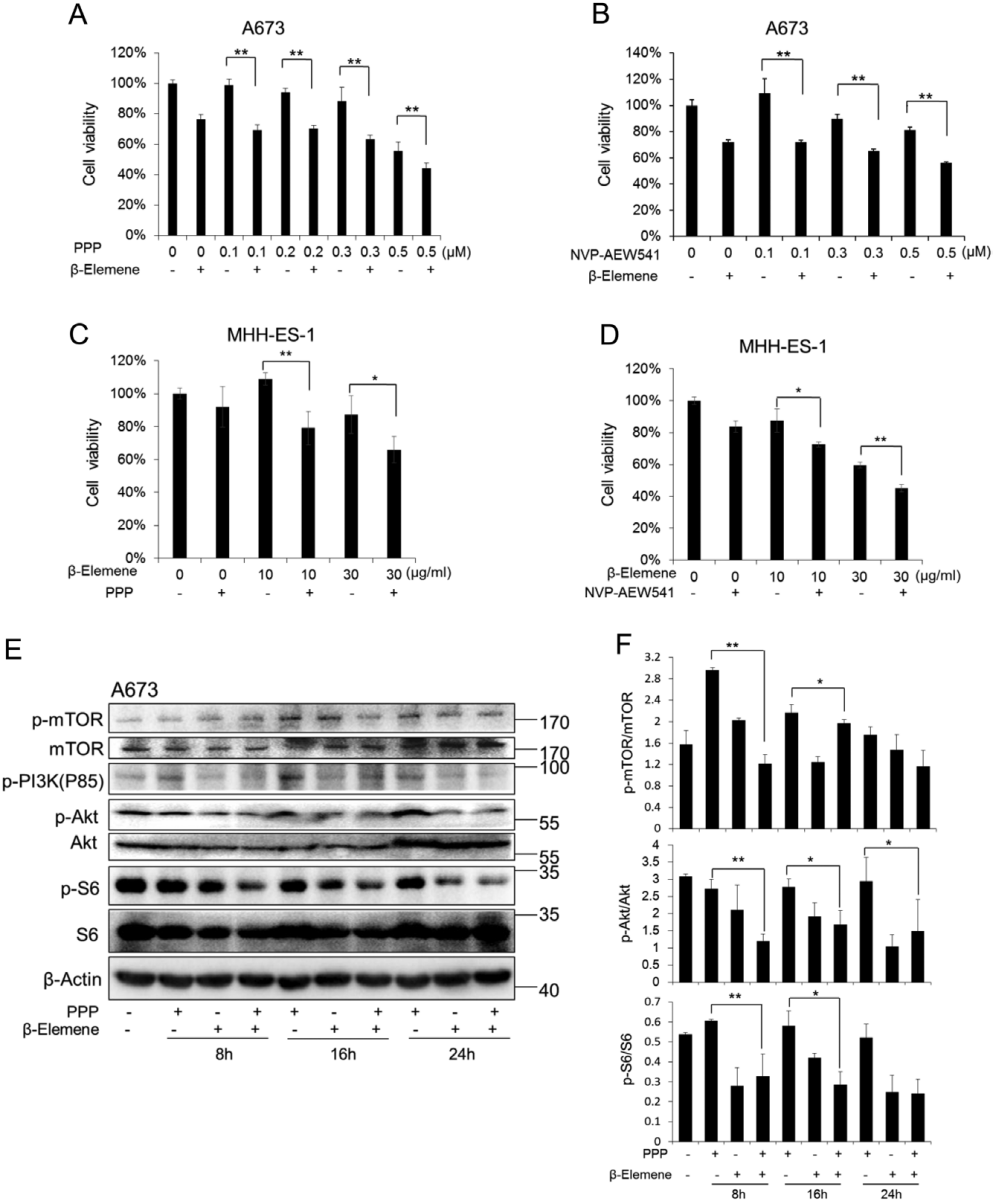
Effects of β-elemene in combination with IGF1R inhibitors on the proliferation of ESFT cells and the signaling pathway. The cells were treated with IGF1R inhibitors and increasing concentrations of β-elemene. (A, B, C and D) Cell viability assays 48 h after the treatment. PPP, 0.1 µM (A and C), NVP-AEW541, 0.2 µM (B and D). All the experiments were conducted in triplicate, and three independent experiments were performed. **, *P* < 0.01; *, *P* < 0.05, indicates the significant difference from the particular treated cells as labeled. Bars, s.d. (E) The cells treated with combination of PPP (0.2 µM) and β-elemene (30 µg/mL) were harvested at the indicated time, and the levels of the indicated proteins were analyzed by Western blot. S6, S6 ribosomal protein. (F) The quantification of the phospho-protein from three independent experiments. ** *P* < 0.01 and * *P* < 0.05 indicate the significant difference from the particular treated cells as labeled.

IGF1R inhibitor picropodophyllin (PPP) efficiently blocks IGF1R activity without affecting IR activity (Girnita *et al*. 2004). Another IGF1R inhibitor NVP-AEW541 is 27-fold more potent to IGF1R than IR at cellular level (Garcia-Echeverria *et al*. 2004). In A673 cells, PPP treatment showed a reduction of cell viability in a dose-dependent manner. The treatment with β-elemene alone (30 µg/mL) led to a 23–26% decrease in cell viability compared to the control (Fig. 2A and B). The combination treatment of β-elemene and increasing concentrations of PPP resulted in a significant reduction in cell viability compared with PPP-alone treatment (*P* < 0.01). We also treated A673 cells with β-elemene and NVP-AEW541 and obtained similar inhibitory effects on cell viability (*P* < 0.01). In MHH-ES-1 cells, PPP (0.1 µM) or NVP-AEW541 (0.2 µM) did not show remarkable effects on cell viability, while the combination treatment of β-elemene and PPP or NVP-AEW541 led to a notable decrease in cell viability (*P* < 0.01) (Fig. 2C and D).

Consistent with the previous reports (Zhang *et al*. 2007), PPP stimulated the phosphorylation of PI3K and Akt in A673 cells (Fig. 2E and F). Combination treatment of β-elemene with PPP resulted in a greater decline of PI3K and Akt phosphorylation. In addition, compared with PPP alone, combination treatment of β-elemene with PPP resulted in a greater decline in the phosphorylation of mTOR and S6 ribosomal protein representing the activity of mTOR.

### β-elemene represses the insulin-driven cellular proliferation of ESFT cells and other tumor cells

**Figure 3:**
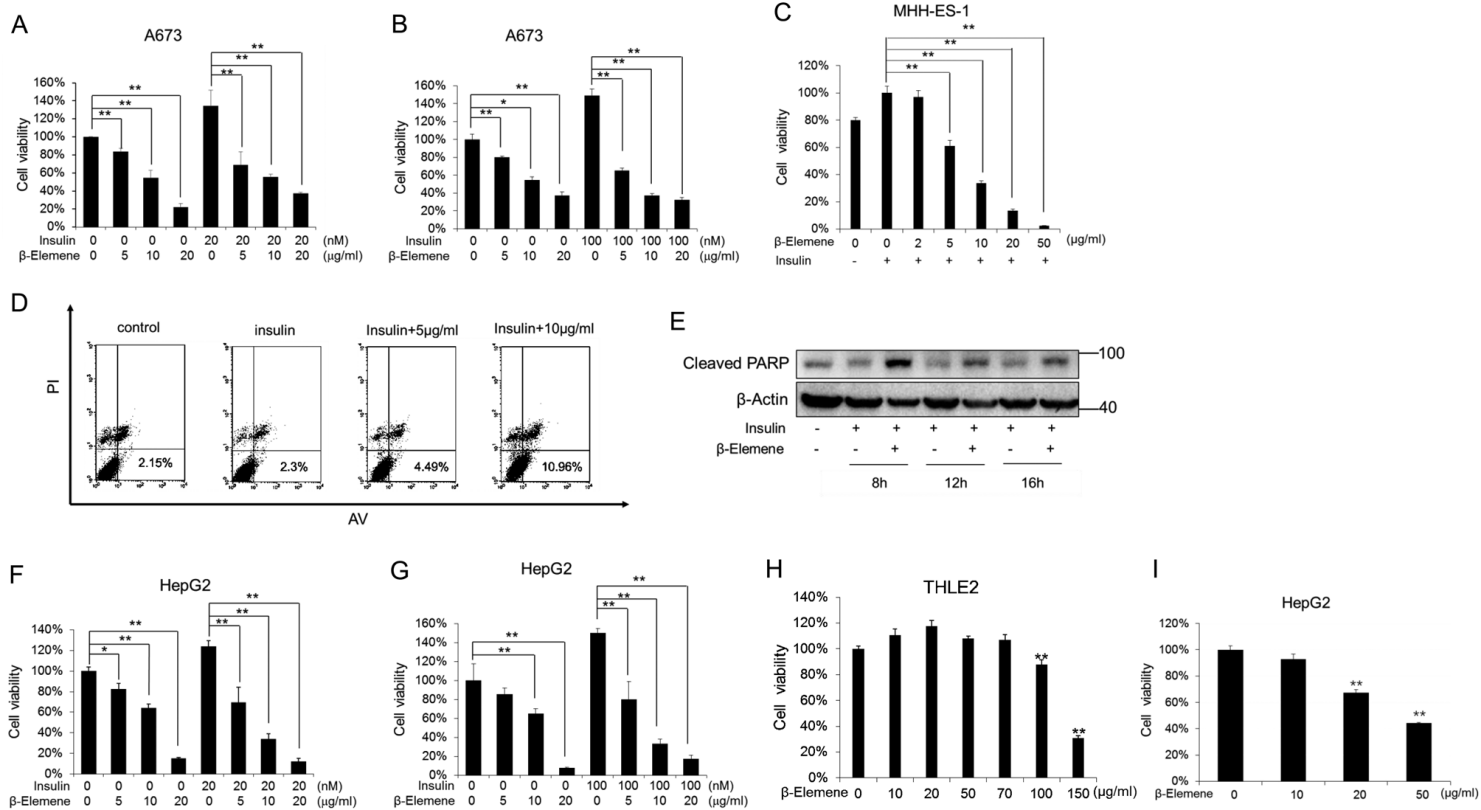
Effects of β-elemene on the insulin-induced cellular proliferation. The cells were treated with or without (−) 20 nM (A and F) or 100 nM (B, C, D, E and G) of insulin and β-elemene after 24 h starvation. (A, B and C) Cell viability assays were performed 48 h after the treatment. (D) Twenty four hours later, the cells were analyzed by flow cytometry after stained by annexin V (AV) and PI. (E) The cells were harvested at the indicated time, and the levels of cleaved PARP were analyzed by Western blot. (F and G) Cell viability assays were performed 48 h after the treatment. (H and I) Cell viability assays for THLE2 and HepG2 cells. All the experiments were conducted in triplicate, and three independent experiments were performed. **, *P* < 0.01, indicates the significant difference from the particular treated cells as labeled (A, B, C, F and G) or the untreated cells (H and I). Bars, s.d.

Since IGF1R blockade treatment results in the activation of IR signaling pathway (Zhang *et al*. 2007, Garofalo *et al*. 2011, Lamhamedi-Cherradi *et al*. 2016) (Fig. 2E), and β-elemene enhanced IGF1R inhibitor’s repression on cell viability (Fig. 2A, B, C and D), we hypothesize that β-elemene may inhibit the activation of IR signaling pathway induced by IGF1R blockade. To clarify this issue, we next investigated the effects of β-elemene on the insulin-driven cellular proliferation of ESFT cells. After the starvation, insulin (20 nM or 100 nM) stimulated the cell growth to 1.4-fold or 1.5-fold compared with control in A673 or MHH-ES-1 cells. Simultaneously these cells were treated with increasing concentrations of β-elemene. β-elemene remarkably inhibited the exogenous insulin-driven ESFT cell growth dose dependently (P<0.01), and the insulin-driven cell growth was downregulated to the level of that without insulin treatment by 5 µg/mL of β-elemene. (Fig. 3A, B and C). The data suggest that the insulin-driven cell growth is sensitive to β-elemene treatment in ESFT cells.

To clarify whether the repression on the insulin-driven cell growth by β-elemene is due to apoptosis, we treated A673 cells with insulin and β-elemene, and stained the cells with PI/Annexin V. Insulin treatment did not change the ratio of the PI^-^/Annexin V^+^ cells, whereas β-elemene (5 and 10 µg/mL) increased the ratio of the PI^-^/Annexin V^+^ cells from 2.3 to 4.49% and 10.96%, respectively (Fig. 3D). The level of cleaved PARP, an apoptosis marker, was decreased by insulin stimulation and also increased remarkably by β-elemene treatment (Fig. 3E). These data suggest a possibility that β-elemene induces apoptosis.

We further investigated the anti-proliferative effects of β-elemene in hepatocellular carcinoma cell HepG2, and β-elemene treatment also led to significant reduction of the insulin-driven cell growth in a dose-dependent manner (*P* < 0.01) (Fig. 3F and G). To examine whether β-elemene is toxic to normal cells, we selected an insulin-responsible normal cell, a normal hepatocyte cell line THLE2 to study the effects. β-elemene treatment did not show any proliferation repression in THLE2 cells even at 50 µg/mL, which is comparative to the IC50 of β-elemene in ESFT cells and HepG2 cells. THLE2 cells only showed a 10% drop in cell viability in response to 100 µg/mL of β-elemene, whereas A673 cells exhibited an approximate 95% reduction (Figs 1A, B and 3H, I). Hence, it is in a tumor-specific manner that β-elemene may inhibit cellular proliferation.

### β-elemene inhibits IR phosphorylation and the downstream signaling specifically in tumor cells

**Figure 4:**
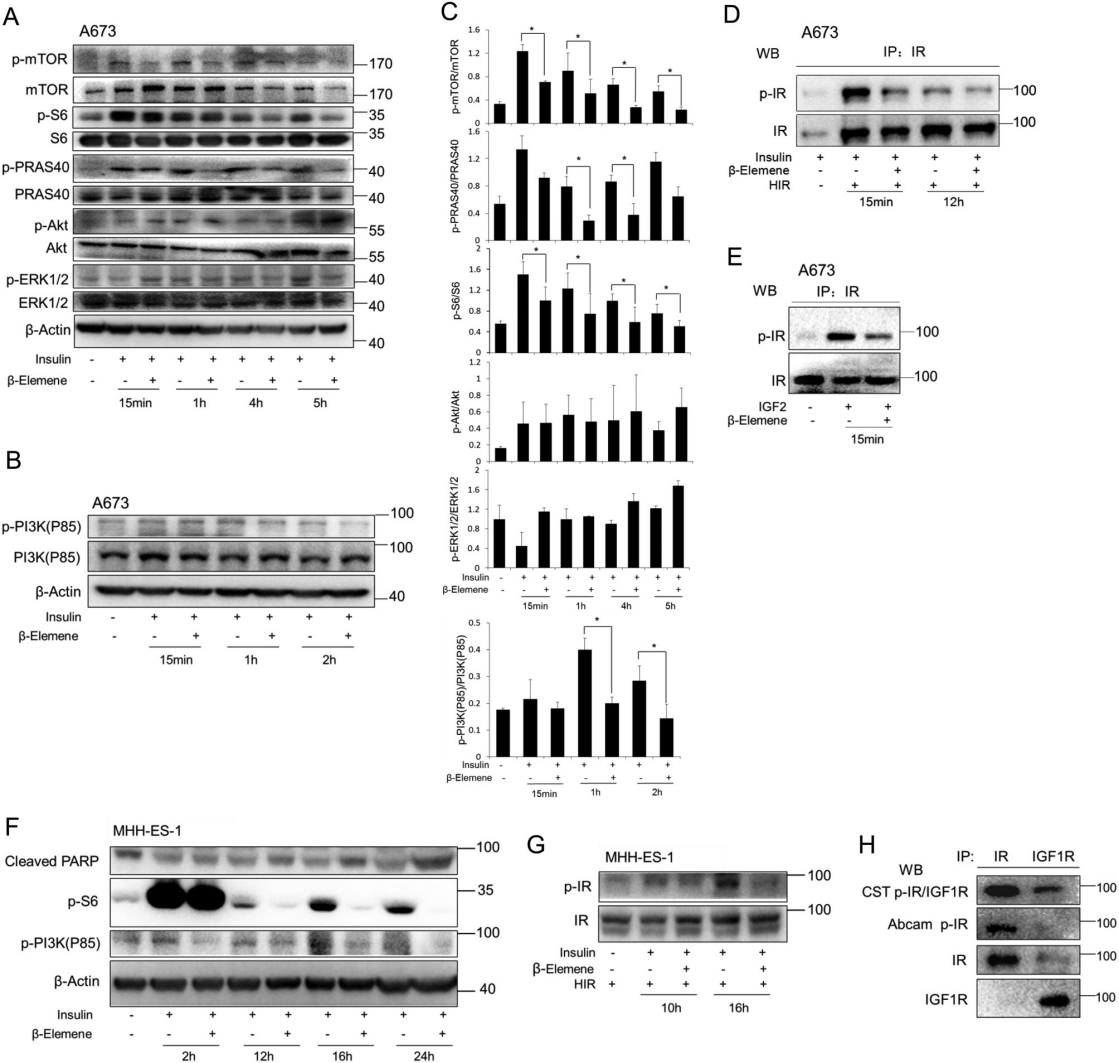
Effects of β-elemene on the phosphorylation of insulin pathway signaling factors in ESFT cells. A673 cells (A, B, C, D and E) were treated with or without (−) insulin (100 nM) (A, B, C and D), or IGF2 (100 ng/mL) (E) together with β-elemene (10 µg/mL) after 24 h starvation. MHH-ES-1 cells (F and G) were treated with or without (−) insulin (100 nM) and β-elemene (10 µg/mL) after 24 h starvation. Human IR (HIR) expression plasmid was introduced into A673 (D) or MHH-ES-1 (G) cells. The cells were harvested at the indicated time, and the levels of the indicated proteins were analyzed by Western blot. (C) The quantification of phospho-protein from three independent experiments. *, *P* < 0.05, indicates the significant difference from the particular treated cells as labeled. (H) A673 cells were harvested and applied to immunoprecipitation with anti-IR or anti-IGF1R antibody, followed by the Western blot with p-IGF1R (Y1135/1136)/p-IR (Y1150/1151) (Cell Signaling) or p-IR (Y1361, Abcam) antibody. S6, S6 ribosomal protein.

We next investigated the effects of β-elemene on the activation of signaling pathways in response to insulin stimulation (Fig. 4). When we treated A673 cells with β-elemene together with insulin, β-elemene treatment decreased notably the phosphorylation of S6 ribosomal protein, PI3K and PRAS40 but not ERK1/2 and Akt stimulated by insulin 4 h after the treatment. The phosphorylation repression by β-elemene was maintained at least till 24 h after the treatment (Fig. 4A, B, C and D). That the activation of both mTOR and PI3K pathways is inhibited by β-elemene treatment implicates a possibility of an activation inhibition to a common upstream factor of these two pathways. Consequently, we studied the effects of β-elemene treatment on IR phosphorylation. The expression plasmid of human IR-B was transfected into A673 cells to construct an IR-hyper-expression model, and the cells were treated with insulin or/and β-elemene after the starvation. We performed the immunoprecipitation with anti-IR antibody followed by the western blot with anti-p-IR antibody. A remarkable downregulation of IR phosphorylation was verified in β-elemene-treated cells (Fig. 4D). To investigate the effects of β-elemene on the phosphorylation of IR-A, we treated the cells with IGF2. The level of phosphorylated IR significantly upregulated by IGF2 was notably downregulated by β-elemene treatment (Fig. 4E). Another anti-p-IR (Y1361, Abcam) antibody has been recently used to recognize p-IR in several other studies (Ilatovskaya *et al*. 2015, Kruger *et al*. 2015, Brenachot *et al*. 2017), which was selected for the next experiments after confirming its specificity (Fig. 4H). In MHH-ES-1 cells, similar results have been obtained. The activation of mTOR and PI3K, and the phosphorylation of IR in response to insulin stimulation were inhibited by β-elemene treatment. Meanwhile, the level of cleaved PARP decreased by insulin stimulation was increased by β-elemene treatment (Fig. 4F and G).

**Figure 5:**
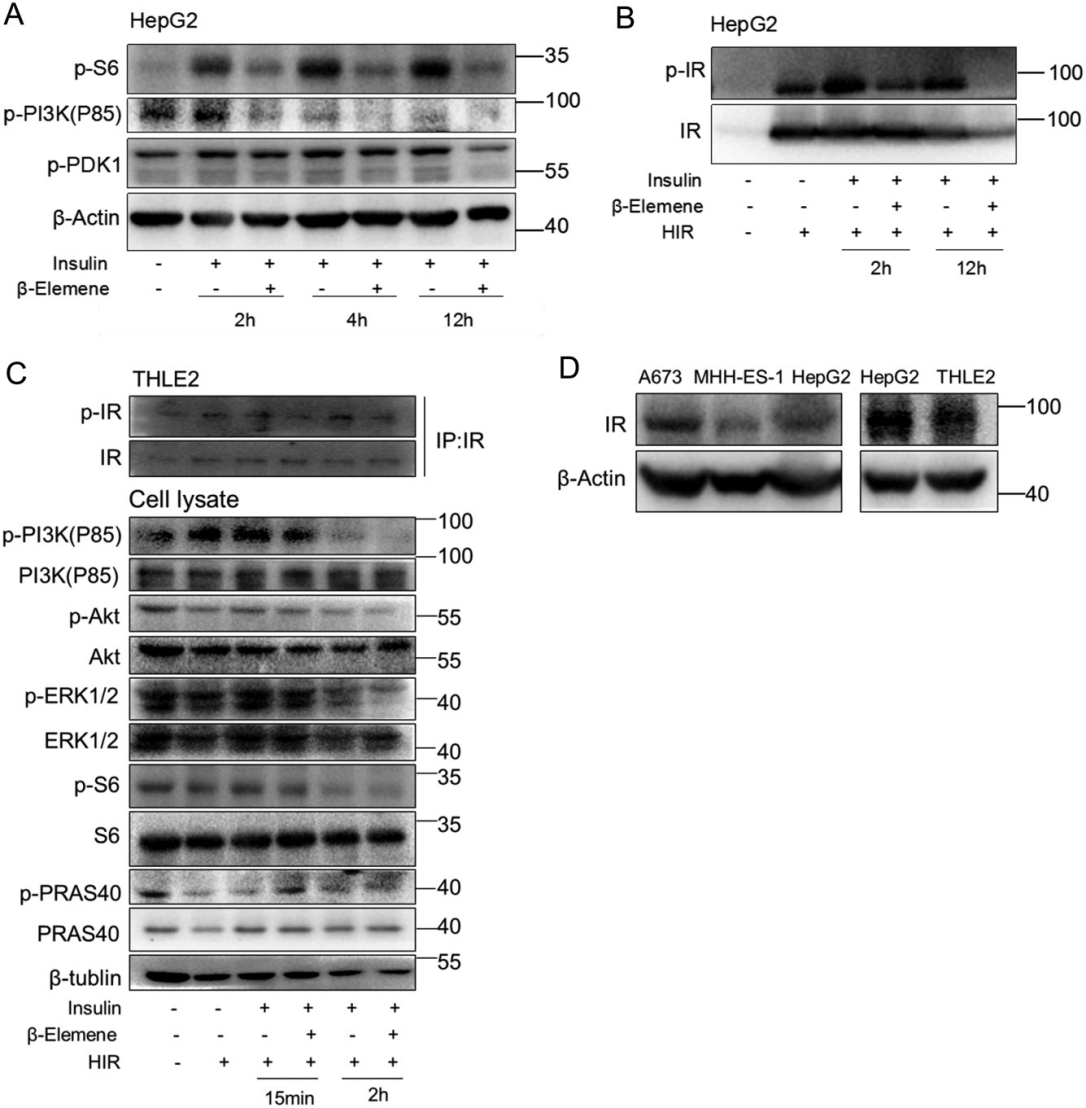
Effects of β-elemene on the phosphorylation of insulin pathway signaling factors in tumor cells and normal hepatocytes. HepG2 (A and B) and THLE2 (C and D) cells were treated with or without (−) insulin (100 nM) and β-elemene (10 µg/mL) after 24 h starvation. Human IR (HIR) expression plasmid was introduced into HepG2 (B) or THLE2 (C) cells. The cells were harvested at the indicated time, and applied to immunoprecipitation with anti-IR antibody followed by Western blot (C) or directly applied to Western blot (A and B). (D) Western blot for endogenous IR. S6, S6 ribosomal protein.

We also tested whether β-elemene could repress the activation of insulin signaling pathway in melanoma cell A2058 and hepatocellular carcinoma cell HepG2. The results showed that the phosphorylation of S6 ribosomal protein, PDK1, PRAS40 and IR was inhibited greatly, and the level of cleaved PARP was increased by β-elemene treatment (Fig. 5A, B and Supplementary Fig. 1, see section on supplementary data given at the end of this article).

Due to the great repression of β-elemene on IR phosphorylation and the downstream signaling, it should be very important to verify whether there is an impairment in normal cells. Next, we treated THLE2 cells with insulin or/and β-elemene after the starvation. Surprisingly, the phosphorylation of IR, S6 ribosomal protein, PI3K, Akt and PRAS40 was not decreased at all in β-elemene-treated cells compared with the control cells (Fig. 5C). These data suggest that β-elemene inhibits IR phosphorylation and the downstream signaling in a tumor-specific manner, which may be due to the hyper-expression of IR in tumor cells (Fig. 5D).

### β-elemene inhibits the tumor growth in ESFT xenograft models

**Figure 6:**
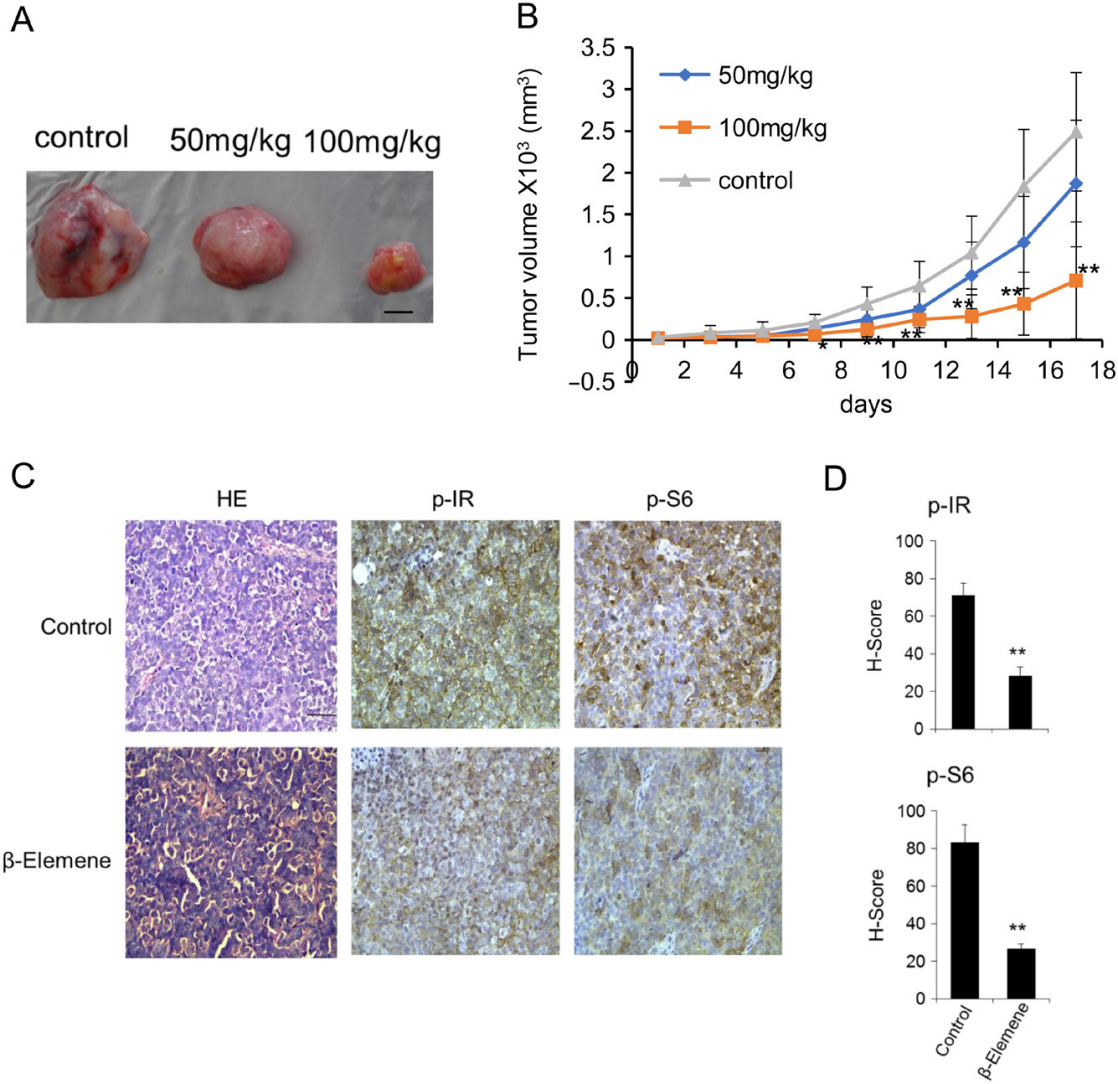
Effects of β-elemene on ESFT growth *in vivo*. The mice bearing A673 xenografts were treated with increasing concentrations of β-elemene. Eight xenografts were involved for each dose. (A) On day 18, tumors were dissected and obtained. Representative images are presented. Scale bar, 5 mm. (B) Tumor volumes were recorded every 2 days. **P < 0.01; *P < 0.05 indicate the significant difference from the control mice. (C) Immunohistochemical analysis of the p-IR and p-S6 ribosomal protein levels and hematoxylin and eosin staining in tumor samples treated with or without (control) β-elemene (100 mg/kg). Scale bar, 50 μm. (D) Quantification of p-IR and p-S6 ribosomal protein from three independent samples. **, *P* < 0.01, indicates the significant difference from the control mice. A full colour version of this figure is available at https://doi.org/10.1530/ERC-18-0370.

Based on the *in vitro* results, we further explored the possibility that β-elemene inhibits the growth of ESFT xenografts *in vivo*. Treatment of the mice bearing A673 xenografts with β-elemene at dose levels of 50 or 100 mg/kg resulted in tumor growth inhibition of 25 or 72% compared with the control (Fig. 6A and B).

Moreover, immunohistological analyses indicated that anti-p-IR or anti-p-S6 ribosomal protein antibody staining of tumor tissues from mice of the β-elemene (100 mg/kg) groups was considerably weaker compared with the control groups (Fig. 6C and D). These data indicate that β-elemene treatment significantly suppresses the tumor growth of ESFT xenografts *in vivo* by inhibiting IR phosphorylation and the downstream signaling.

## Discussion

ESFTs are highly malignant tumors, and conventional treatments did not bring approving results. β-elemene is effective against a wide variety of tumors (Ding *et al*. 2013, Liu *et al*. 2014, Zhu *et al*. 2014, Zhao *et al*. 2015, Wu *et al*. 2017), whereas there was no evidence demonstrating the potential of β-elemene in controlling ESFTs before. We show here that β-elemene suppresses the proliferation of ESFT cells and xenografts and induces the apoptosis of ESFT cells (Figs 1, 2, 3, 4 and 6).

Recent studies concerning novel treatments of ESFTs focus on targeting therapies and immunotherapies. Among these therapies, IGF1R blockade is considered to be effective toward ESFTs in clinical trials (Kurzrock *et al*. 2010, Olmos *et al*. 2010). Unfortunately, many patients develop resistance to the therapy and disease recurrence (Toretsky & Gorlick 2010). IR-A is reported to be expressed more than IR-B isoform in ESFT cells (Garofalo *et al*. 2011). Since IGF2 is produced by ESFT cells, resistance to IGF1R therapy is believed to be driven by an IGF2/IR-A loop (Zhang *et al*. 2007, Hendrickson & Haluska 2009, Garofalo *et al*. 2011). Therefore, suppression of IR signaling simultaneously combined with IGF1R blockade treatment could be expected. For example, IGF1 blockade becomes more effective to ESFTs in combination with mTOR inhibitors (Zhong *et al*. 2014) and PI3K inhibitors (Anderson *et al*. 2015). Our results show that β-elemene inhibits both IGF1/2- and insulin-driven cellular proliferation (Figs 1F, H and 3A, B, C, F, G), and β-elemene represses the activation of both IR-A and IR-B (Fig. 4D and E), the possible causes of IGF1R blockade resistance. In combination of IGF1R inhibitors, β-elemene enhances the antitumor activities of IGF1R inhibitors in ESFT cells and represses the activation of mTOR and PI3K (Fig. 2). It is anticipated that β-elemene in combination with IGF1R inhibitors should be effective to IGF1 signaling hyperactivated tumors by targeting both IR phosphorylation and IGF1 signaling.

Several studies have demonstrated the mechanisms involved in the antitumor effects of β-elemene (Li *et al*. 2013,^ 2016^, Liu *et al*. 2014, Zhao *et al*. 2015, Wu *et al*. 2017), whereas the detailed molecular mechanism remains to be clarified. We show here that β-elemene inhibits the phosphorylation of IR and the downstream factors, including PI3K, PRAS40, mTOR and S6 ribosomal protein specifically in tumor cells (Figs 4, 5 and Table 1). Therefore, IR phosphorylation inhibition could be the important mechanism through which β-elemene suppresses cellular proliferation. Surprisingly, although β-elemene suppressed notably the phosphorylation of Akt induced by IGF1R blockade, the insulin-stimulated phosphorylation of Akt was not inhibited significantly by β-elemene (Figs 2E and 4A). These data imply that the phosphorylation of Akt might be controlled by other upstream signaling besides IR signaling. In addition, tumor cells were also reported to overcome IGF1R inhibition in an EGFR-dependent manner (Desbois-Mouthon *et al*. 2009), indicating that EGF signaling could be another way mediating IGF1R blockade resistance. To clarify this possibility, we examined the alteration of ERK1/2 phosphorylation. However, ERK1/2 phosphorylation was not upregulated after IGF1R blockade treatment, and β-elemene treatment did not show any suppression on ERK1/2 phosphorylation (Fig. 4A).

**Table 1:** The results of the alteration of the protein phosphorylation in different cell lines.

Cell lines	p-IR	p-PI3Kp85	p-PRAS40	p-mTOR	p-S6	p-Akt	p-ERK
A673	↓	↓	↓	↓	↓	–	–
MHH-ES1	↓	↓	NA	NA	↓	NA	NA
HepG2	↓	↓	NA	NA	↓	NA	NA
A2058	↓	↓	↓	NA	↓	NA	NA
THLE2	–	–	–	NA	–	–	–

↓, decreased; –, not changed; NA, not done.

IR phosphorylation results in different responses in different types of cells. A major consequence of IR activation in liver is the inhibition of gluconeogenesis and the activation of glycogen storage, whereas in epithelial cells is the stimulation of proliferation and the inhibition of apoptosis (Venkateswaran *et al*. 2007, Algire *et al*. 2011). Targeting IR results in the growth repression of tumor cells (Chan *et al*. 2016). Since the amplification and mutation of IR are rare in tumors (Pollak 2012), and IR signaling plays a crucial role in glucose metabolism, the IR blockade is always considered too dangerous to be used against tumors. In addition, the compensatory hyperinsulinemia caused by PI3K or mTOR inhibitors has been observed due to the signaling inhibition in normal tissues (Hernandez-Fisac *et al*. 2007, Chandarlapaty *et al*. 2011). Surprisingly, besides ESFT cells, β-elemene could inhibit the insulin-driven proliferation and IR phosphorylation in hepatocellular carcinoma and melanoma cells (Fig. 5B and Supplementary Fig. 1B). However, β-elemene does not show any repression effects on the growth of normal heptocytes at the toxic concentrations in ESFT cells and hepatocellular carcinoma cells (Figs 1A, B and 3H, I). Further, β-elemene inhibits neither IR phosphorylation nor the downstream signaling in normal hepatocytes (Fig. 5C and D). Thus, β-elemene provides a possible way to target IR phosphorylation in tumor cells without changing the IR signaling in normal cells. Patients with type 2 diabetes or obesity have modestly increased cancer risk and/or cancer prognosis (Calle *et al*. 2003, Ma *et al*. 2004,^ 2008^, Giovannucci *et al*. 2010, Algire *et al*. 2011). This may be at least partly attributable to the exposure to hyperinsulinaemia. Therefore, targeting IR or its downstream signaling could be considered to treat this kind of patients. It may be possible to use β-elemene to target IR phosphorylation in cancer patients with diabetes or obesity because of the low adverse effects of β-elemene on IR signaling in normal cells.

However, the mechanism of β-elemene targeting IR phosphorylation in detail remains largely unknown. Since these effects of β-elemene are tumor specific, and the amplification and mutation of IR are rare in tumors, we do not consider for a direct phosphorylation repression of IR by β-elemene. The deficiency of various phosphatases in tumor has been reported (Kleppe *et al*. 2010, Shields *et al*. 2013, Le Sommer *et al*. 2018); thus, we hypothesize that β-elemene might induce the expression of IR-associated phosphatases in tumor cells, resulting in the suppression of IR phosphorylation. More studies are needed to clarify this issue. Taken together, our results show that β-elemene targets IR phosphorylation and the downstream signaling to inhibit ESFT growth and enhance the effects of IGF1R inhibitors on ESFT cells. This study highlights the potential for optimizing therapeutic strategies of ESFTs by β-elemene and provides evidence for novel approaches by β-elemene alone or in combination with IGF1R blockade in ESFTs and IR signaling hyperactivated tumors.

## Supplementary Material

Supporting Figure 1

## Declaration of interest

The authors declare that there is no conflict of interest that could be perceived as prejudicing the impartiality of the research reported.

## Funding

This work was supported by the Climbing Scholars Supporting Program of Liaoning Province, Natural Science Foundation of Liaoning Province (2014023039), National Natural Science Foundation of China (81772971) and Liaoning Provincial program for Top Discipline of Basic Medical Sciences.

## Author contribution statement

D W, D L and L H designed research; D W, D L, T Z, L G, F M, C Z and L H performed the experiments and analyzed the data; G L analyzed the data; L H wrote the manuscript. All authors read and approved the manuscript.
