# Supplementary material for: Antitumor effects of β-elemene via targeting the phosphorylation of insulin receptor
Source: Endocr Relat Cancer. 2018 Nov 12;26(2):187–99. doi: 10.1530/ERC-18-0370 (PMC6347285; doi:10.1530/ERC-18-0370)
Supplement: Supporting Figure 1 [file supplementary_figure_1.pdf]

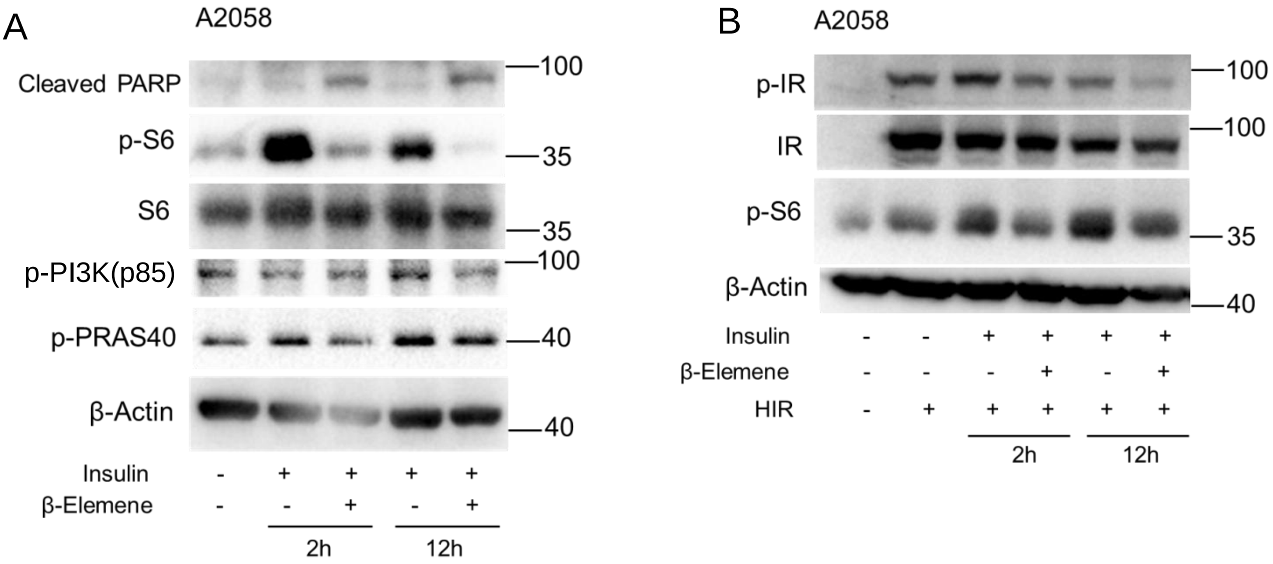

Supplementary Fig. 1

Supplementary Fig. 1 Effects of β-elemene on the phosphorylation of insulin pathway signaling factors in melanoma cells. A2058 cells were treated with or without (-) insulin (100nM) and β-elemene (10μg/ml) after 24h starvation. Human IR (HIR) expression plasmid was introduced into cells (B). The cells were harvested at the indicated time, and the levels of the indicated proteins were analyzed by Western blot. S6, S6 ribosomal protein.
